# Pharmacodynamic Mechanisms of Cicadae Periostracum in Parkinson’s Disease: A Metabolomics-Based Study

**DOI:** 10.3390/ijms26020544

**Published:** 2025-01-10

**Authors:** Mengmeng Li, Fuyu Xiong, Shifei Wu, Wenlong Wei, Hanze Wang, Yajun Qiao, Dean Guo

**Affiliations:** 1 College of Pharmaceutical Sciences, Changchun University of Chinese Medicine, Changchun 130117, China; lmm128063@163.com (M.L.); xiongfuyu2001@163.com (F.X.); wanghanze_123@163.com (H.W.); 2 Shanghai Research Center for Modernization of Traditional Chinese Medicine, National Engineering Research Center of TCM Standardization Technology, Shanghai Institute of Materia Medica, Chinese Academy of Sciences, Shanghai 201203, China; wushifei@simm.ac.cn (S.W.); weiwenlong@simm.ac.cn (W.W.); qiaoyajun@simm.ac.cn (Y.Q.)

**Keywords:** Parkinson’s disease, Cicadae Periostracum, metabolomics, oxidative stress, mechanisms of action

## Abstract

Cicadae Periostracum (CP) is a traditional Chinese animal-derived medicine with the potential to treat Parkinson’s disease (PD). This study aims to explore the pharmacodynamic mechanisms of CP against PD-based on metabolomics technology and provide a theoretical basis for developing new anti-PD medicine. First, MPP^+^-induced SH-SY5Y cells were used to evaluate the anti-PD activity of CP. In the animal study, an MPTP-induced PD mouse model was employed to assess CP’s therapeutic effects. Immunofluorescence (IF) staining and Western blotting (WB) were used to evaluate its neuroprotective activity on neurons. A Serum metabolomics analysis was conducted to examine CP’s regulatory effects on metabolites and to identify vital metabolic pathways. Finally, cellular experiments were performed to validate the critical pathways. Cellular activity experiments demonstrated that CP mitigates MPP^+^-induced SH-SY5Y cytotoxicity, inhibits apoptosis, and restores mitochondrial homeostasis. Animal experiments revealed that CP significantly alleviates dyskinesia in PD mice, enhances motor performance, and restores neuronal integrity while reducing α-synuclein (α-syn) aggregation in the striatum (STR), showing its strong anti-PD effect. Metabolomic analysis revealed that CP can significantly improve the metabolic disorders of ten biomarkers that are mainly involved in amino acid metabolism and fatty acid β-oxidation and are closely related to oxidative stress pathways. Finally, pathway verification was performed, and the results show that CP exerted neuroprotective effects against PD through the dual signaling pathways of Bcl-2/Bax/Caspase-3 and Nrf2/HO-1. This study provides a comprehensive strategy for elucidating the mechanisms by which CP exerts its therapeutic effects against PD, highlighting its potential in developing anti-PD drugs.

## 1. Introduction

PD is a progressive neurodegenerative disorder clinically characterized by muscle rigidity, resting tremor, and bradykinesia [1]. The main pathological features of PD include the selective loss of dopmainergic neurons in the midbrain substantia nigra (SN) and the presence of pathological markers such as Lewy bodies (LB) in the cytoplasm of the remaining dopaminergic neurons [2]. It is widely accepted that the primary pathogenic mechanisms of PD include oxidative stress, apoptosis, inflammation, mitochondrial dysfunction, and α-syn aggregation [3]. With its increasing prevalence, PD has become one of the most common diseases in the elderly, following cancer and cardiovascular diseases [4]. Despite significant advances in understanding the pathophysiology of PD, there remains no cure. Dopamine (DA) replacement therapies can alleviate symptoms, but their efficacy diminishes over time [5], often accompanied by side effects. More effective and less toxic therapeutic options are urgently needed. Therefore, it is crucial to explore potential treatments from traditional medicine.

Traditional Chinese medicine (TCM) has been a valuable resource in China for thousands of years. Due to its multi-component and multi-target properties, TCM offers promising avenues for discovering novel treatments for PD [6]. Animal-derived medicines, an integral component of TCM, have a long history of medicinal use in China [7]. Cicadae Periostracum (CP), the cast-off skin of Cryptotympana pustulate Fabricius, a commonly used traditional animal medicine, contains chitin, amino acids, *N*-acetyldopamine, and proteins, among other components [8], and exhibits diverse pharmacological activities, including antioxidant, anti-inflammatory, antitussive, and antispasmodic effects. Research has identified *N*-acetyldopamine as one of the essential bioactive compounds in CP, with antioxidant and anti-inflammatory properties, potentially contributing to its therapeutic effects [9,10]. Studies have found that CP extract could effectively improve PCPA-induced insomnia in rats and significantly affect the expression of 5-HT1A, BDNF, and DARPP-32 proteins [11]. Other studies have demonstrated that CP water extracts primarily modulate neurotrophic factors through the nurr1 signaling pathway, neuroinflammation, and mitochondrial-mediated apoptosis, promoting neuroprotective signaling [12]. This positions CP as a promising candidate for the treatment of PD.

Scientific evidence indicates that metabolic changes may precede and directly contribute to neurodegeneration [13]. Metabolomics is an effective tool for elucidating the therapeutic mechanisms of TCM by identifying changes in metabolites and metabolic pathways and highlighting critical pathways for further validation [14]. It is beneficial in studying complex pathophysiology in general and in PD specifically [15]. To our knowledge, in this study, we first used non-targeted serum metabolomics to explore the mechanisms of CP’s action in treating PD. The findings highlight the potential of CP in PD treatment, providing new insights into therapeutic strategies for PD.

## 2. Results

### 2.1. Cell Experiment Results

#### 2.1.1. CP Can Improve MPP^+^-Induced Cytotoxicity

**Figure 1:**
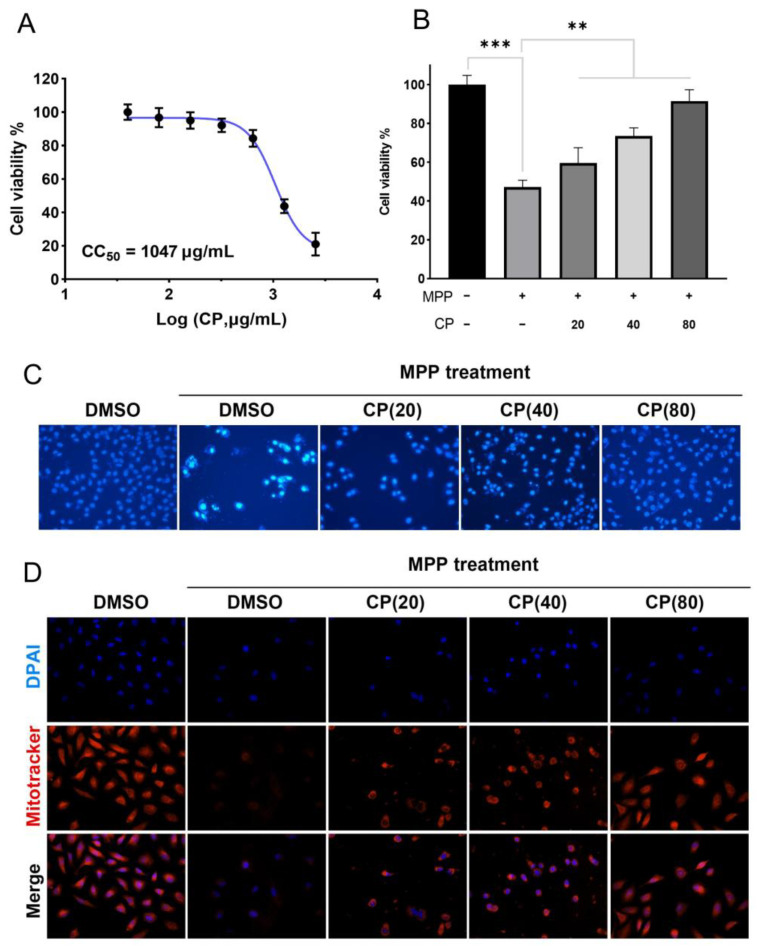
CP autotoxicity test and protective effect on MPP^+^-induced SH-SY5Y cells; CP reduces apoptosis and protects mitochondrial homeostasis. (**A**) Cytotoxicity assessment of CP in SH-SY5Y cells. (**B**) Neuroprotective effect of CP against MPP^+^-induced cytotoxicity in SH-SY5Y cells. (**C**) Representative images of IF staining; CP reduces MPP^+^-induced cell apoptosis. Scale bar, 50 μm. (**D**) Representative images of IF staining; CP restores MPP^+^-induced mitochondrial homeostasis in SH-SY5Y cells. Scale bar, 20 μm. ** *p* < 0.01, and *** *p* < 0.001 compared with the model group.

A CCK-8 assay was conducted to evaluate the cytotoxicity of CP. It was observed that CP had a negligible impact on cell viability, as shown in Figure 1A (CC50 ≈ 1047 μg/mL). To ascertain the therapeutic efficacy of CP, SH-SY5Y cells were pre-incubated with MPP^+^ (0.8 mM) for 24 h, followed by a 24 h treatment with varying concentrations of CP. We observed that CP at concentrations of 40 μg/mL and 80 μg/mL significantly reversed the MPP^+^-induced decrease in cell viability (Figure 1B). These results suggest that CP possesses a protective effect against MPP^+^-induced cytotoxicity in SH-SY5Y cells.

#### 2.1.2. CP Can Reduce MPP^+^-Induced Apoptosis in SH-SY5Y Cells

In this study, we investigated the effects of CP on MPP^+^-induced apoptosis in SH-SY5Y cells using Hoechst 33342 staining. The Hoechst staining results demonstrate that CP at a concentration of 80 μg/mL significantly mitigated MPP^+^-induced apoptosis in SH-SY5Y cells and restored their morphological integrity (Figure 1C).

#### 2.1.3. CP Restores Mitochondrial Membrane Potential of SHSY5Y Cells Induced by MPP^+^

Mitochondrial homeostasis is closely associated with apoptosis. In this study, we investigated the effects of CP on mitochondria homeostasis in MPP^+^-induced SH-SY5Y cells. The mitochondrial staining results reveal that the mitochondrial membrane potential was significantly reduced after MPP^+^ treatment. CP treatment markedly increased the mitochondrial membrane potential, restored the abnormal mitochondrial morphology induced by MPP^+^, and improved mitochondrial homeostasis (Figure 1D).

### 2.2. Results of Animal Analysis

#### 2.2.1. Behavioral Analysis of CP in the MPTP-Induced PD Mice Model

**Figure 2:**
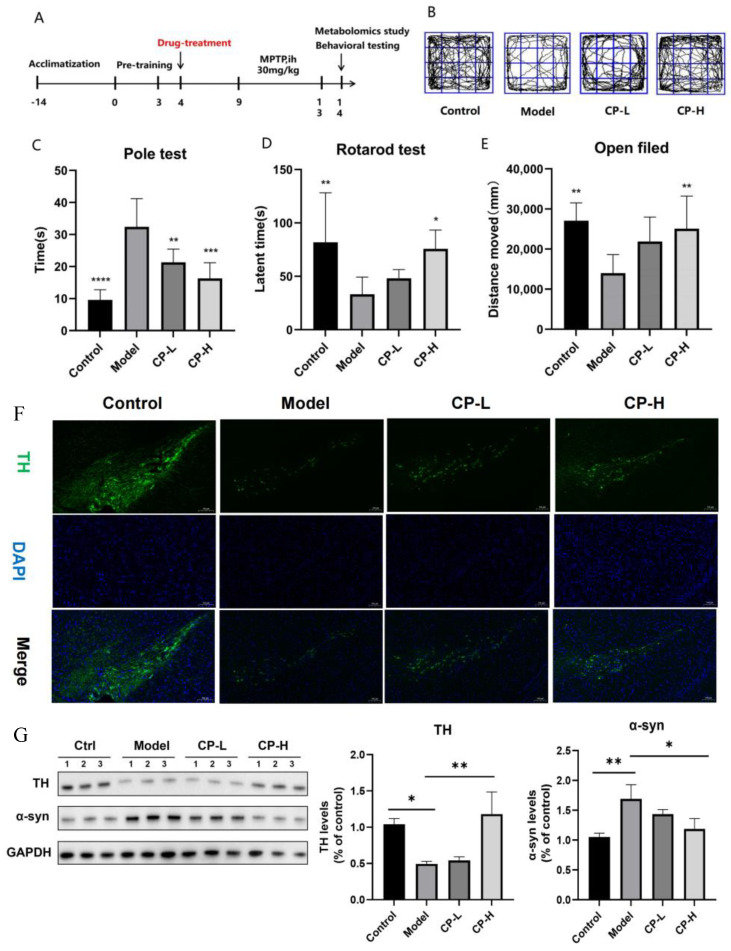
MPTP model establishment, behavioral, IF staining, and WB experimental results. (**A**) MPTP modeling; schematic diagram of drug treatment time. (**B**) The mice activity trajectory in the open field test (*n* ≥ 8). (**C**) Pole test results. (**D**) Rotarod test result. (**E**) Open field test results. (**F**) Representative micrographs of TH IF staining in the SN; scale bar-250 μm. (**G**) WB analysis of TH and α-syn in the midbrain of different groups. Data are expressed as the mean ± standard deviation. * *p* < 0.05, ** *p* < 0.01, *** *p* < 0.001 and **** *p* < 0.0001 compared with the model group.

We started behavioral tests after the last drug treatment, including pole, rotarod, and open field tests, to evaluate the mice’s movement and limb coordination ability [16] (Figure 2C–E). The results show that in the pole test, the climbing time in the model group was significantly longer than the control group; after CP administration, the climbing time was shortened considerably (*p* < 0.001), indicating that CP improved the MPTP-induced bradykinesia of PD mice; in the rotarod test, the model group spent less time on the rotating rod than the control group, while the CP-H group stayed longer (*p* < 0.05), indicating that CP improved the exercise ability of PD mice; in the open field test, the model group spontaneous activity decreased, the CP-H group increased (*p* < 0.01), and the total distance was significantly higher than model group; the activity trajectory of mice in the open field is shown in Figure 2B. The above behavioral results show that CP could improve the movement disorder of PD mice induced by MPTP and improve motor ability.

#### 2.2.2. CP Protects the MPTP-Induced Loss of TH Neurons in the Midbrain and Reduces α-Syn Aggregation in STR

The main characteristics of PD are the loss of dopaminergic neurons in the SN and the aggregation of α-syn to form LB [17]. Therefore, TH and α-syn are vital proteins in the study of the pathogenesis of PD. The expression level and distribution of TH directly reflect the health status of dopaminergic neurons [18]. In this study, we used IF staining to investigate the protective effect of CP on TH neurons in MPTP-induced PD mice; WB was used to detect the effects of CP administration on the TH content in the midbrain and the α-syn content in the STR. IF staining results show that severe TH dopaminergic neuron damage occurred in the SN of mice in the MPTP group, while CP significantly restored TH neuron levels and alleviated TH neuron damage caused by MPTP (Figure 2F). WB results show that, compared to the control group, the TH level in the model group was reduced (*p* < 0.05) and α-syn was significantly increased (*p* < 0.01); after administration of CP, TH levels were significantly increased and the α-syn levels were reduced, indicating that CP can protect mouse TH neurons in the midbrain from damage caused by MPTP and reduce the aggregation of α-syn in the STR (Figure 2G).

### 2.3. Results of Metabolomics Analysis

#### 2.3.1. Multivariate Data Analysis

**Figure 3:**
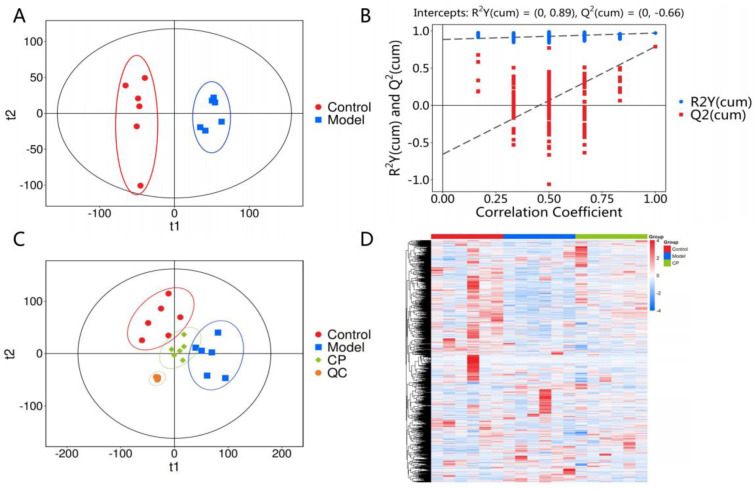
Multivariate data analysis from UPLC-MS/MS. (**A**) OPLS-DA score plots of the control and model groups (*n* = 6). (**B**) OPLS-DA permutation test of the control and model groups. (**C**) PCA score plots of all groups. (**D**) Cluster heat map analysis of differential metabolites in the serum of all groups. The horizontal axis in the figure represents different sample groups, the vertical axis represents all metabolites, and the color blocks at different positions represent the relative expression of metabolites at the corresponding positions. Red indicates a high expression of the substance and blue indicates a low expression.

The typically based peak intensity chromatograms of plasma samples were analyzed in positive and negative modes (Figure S1). There was a significant separation between the model group and the control group in the OPLS-DA score plot (Figure 3A). The OPLS-DA model was validated using a permutation test (Figure 3B). According to R2Y (0.89) and Q2 (−0.66), the model had strong predictive and explanatory abilities. The PCA score plots indicate that the CP and model groups were separated from the control groups. Meanwhile, the QC group was gathered, indicating that the instrument was stable (Figure 3C). The metabolic profile of the CP group was closer to that of the control group than that of the model group, indicating that the metabolic disorder caused by the model was alleviated after CP treatment. Cluster heat map analysis was performed on all samples, and a cluster heat map was drawn (Figure 3D). The results show that CP regulated the content of many metabolites and significantly improved the metabolic disorders of PD mice.

#### 2.3.2. Identification of Endogenous Metabolites

**Figure 4:**
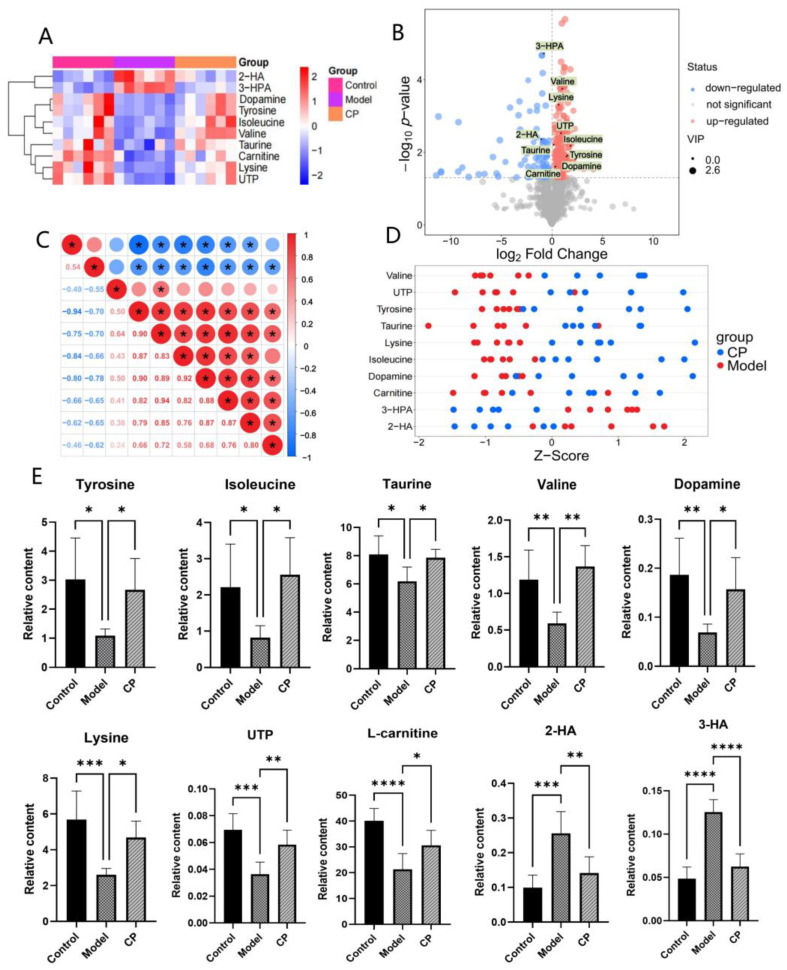
Statistical analysis of differential metabolites. (**A**) Heatmap analysis of differential metabolites in the serum of CP mice. (**B**) Volcano plot of differential metabolites in the serum of mice between the CP and the model group. (**C**) Correlation analysis of differential metabolites in the serum of mice between the CP and the model group. (**D**) Z-score analysis of differential metabolites in the serum of mice between the CP and the model group. (**E**) Histogram of differential metabolites. Comparison of relative contents of the serum differential metabolites in the CP group. * *p* < 0.05, ** *p* < 0.01, *** *p* < 0.001, **** *p* < 0.0001 compared to the model group.

The differential metabolites were screened between the control and model groups according to VIP values (>1.0) and *t*-test (*p* < 0.05). As a result, 19 differential metabolites were screened out (Table 1). Compared to the control group, two differential metabolites (2-Hydroxyoctanoic acid (2-HA), 3-(3-Hydroxyphenyl) propanoic acid (3-HPA)) were significantly increased, and seventeen differential metabolites (serine, threonine, isoleucine, DA, UTP, etc.) were significantly decreased in the model group. CP could regulate ten metabolites (valine, tyrosine, DA, L-carnitine, etc.). To further clarify the distribution of the ten differential metabolites in different groups, we used a cluster heat map analysis (Figure 4A), a volcano plot (Figure 4B), correlation analysis (Figure 4C), and z-score (Figure 4D) analysis, and the relative contents of the ten metabolites were statistically analyzed (Figure 4E). Compared to the model group, these metabolites were significantly regulated, and the metabolic disorders of mice were improved after CP treatment.

#### 2.3.3. Metabolic Pathway Analysis

**Figure 5:**
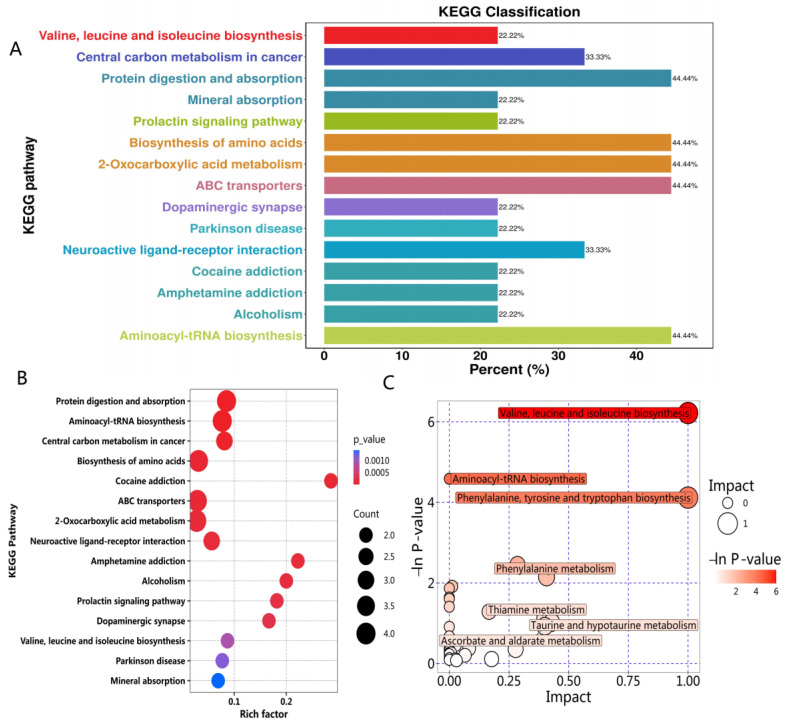

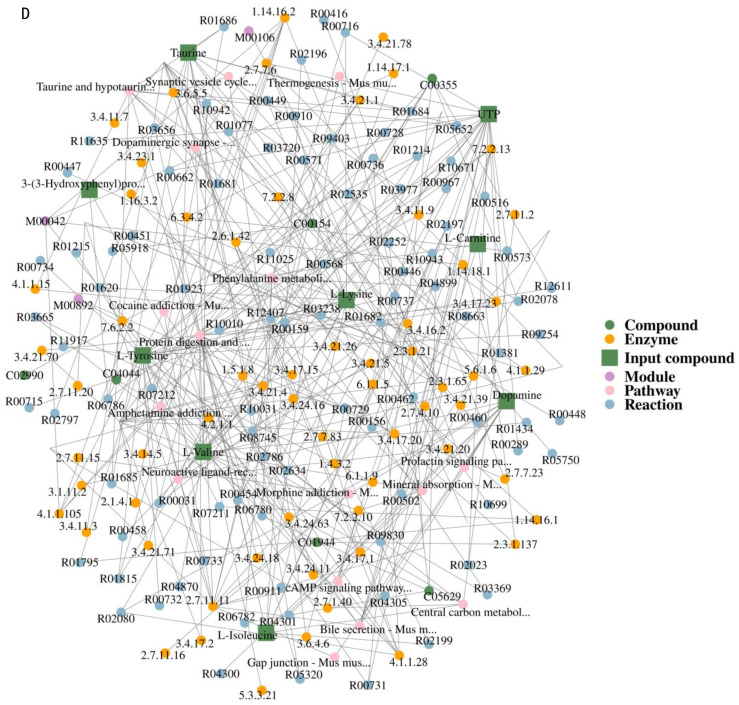
Metabolic regulatory pathways analysis. (**A**) KEGG pathway classification analysis. (**B**) Rich factor analysis. (**C**) Bubble plot impact pathway analysis. (**D**) The regulatory network plot analysis.

The ten differential metabolites were imported into MetaboAnalyst to explore the potential mechanisms of CP against PD [19]. The KEGG pathway classification, rich factor, bubble plot impact pathway, and regulatory network plot analysis are shown in Figure 5. The results show that the metabolic pathways involved in the metabolites of CP-treated PD mice mainly included branched-chain amino acid metabolism, tyrosine metabolism, fatty acid β-oxidation, cysteine and methionine metabolism, etc.; the most critical metabolisms are the amino acid metabolism and lipid metabolism, as these metabolic pathways may be a potential target for the treatment and prevention of PD [20]. The literature shows that the amino acid and lipid metabolism pathways are closely related to oxidative stress [21], and oxidative stress plays a vital role in the degeneration of dopaminergic neurons in PD [22,23]. Therefore, we regard oxidative stress as the critical pathway for CP to treat PD and conduct mechanistic studies in the next step.

### 2.4. Mechanism Analysis of CP Treating PD

#### 2.4.1. CP Reduces MPP^+^-Induced Apoptosis in SHSY5Y Cells via Bax/Bcl-2/Caspase-3 Pathway

**Figure 6:**
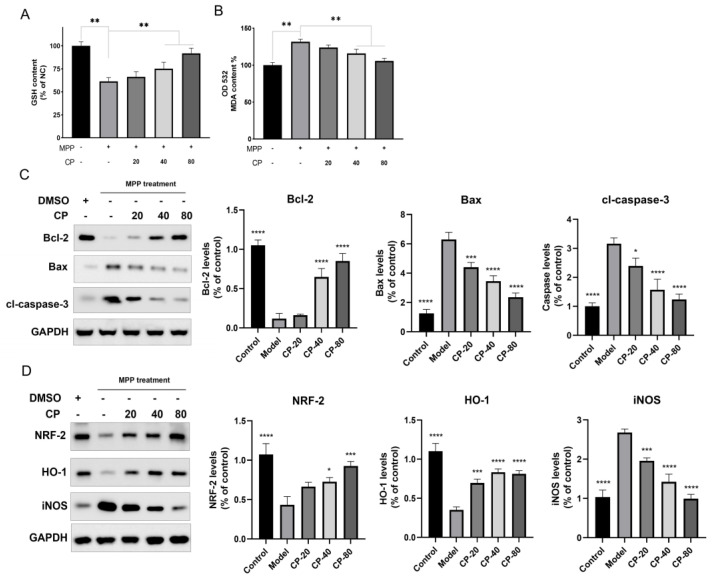
CP reduces apoptosis and oxidative stress in MPP^+^-induced SH-SY5Y cells. (**A**) GSH level in MPP^+^-induced SH-SY5Y cells in different groups. (**B**) MDA level in MPP^+^-induced SH-SY5Y cells in different groups. (**C**) WB analysis of Bcl-2, Bax, and cl-caspase-3 in different groups and statistical analysis (CP, μg/mL). (**D**) WB analysis of Nrf2, HO-1, and iNOS expression in different groups and statistical analysis. Data are expressed as the mean ± standard deviation. * *p* < 0.05 compared with the model group. ** *p* < 0.01; *** *p* < 0.001; **** *p* < 0.0001.

In the previous Hoechst staining study, we found that CP can significantly reduce MPP^+^-induced apoptosis of SH-SY5Y cells and restore their morphological integrity. In the canonical apoptotic pathway, Bax (Bcl-2 Associated X-protein) expression increased while Bcl-2 (BCL2 Apoptosis Regulator) expression decreased, leading to a reduction in mitochondrial membrane potential and the release of cytochrome C, which in turn activates caspase-3 to initiate apoptosis [24]. WB analysis assessed the protein expression levels in different groups. Cells exposed to MPP^+^ treatment exhibited a significant decrease in Bcl-2 protein levels and a marked increase in Bax and cleaved-caspase-3 (cl-caspase-3) protein expression (Figure 6C). Treatment with CP led to a significant restoration of Bcl-2 levels and a considerable reduction in the expression of Bax and caspase-3. These findings indicate that CP attenuates cell apoptosis by inhibiting the Bax/Bcl-2/caspase-3 apoptotic pathway.

#### 2.4.2. CP Reduces MPP^+^-Induced Oxidative Stress in SHSY5Y Cells via Nrf2/HO-1 Pathway

Previous studies found that CP can relieve cell mitochondrial homeostasis and reduce cell apoptosis (Figure 1 and Figure 2). Next, we used GSH and MDA kits to detect each group’s oxidative stress level and WB to detect the level of the related proteins [25]. GSH content assays indicated that, after treatment with CP at 40 μg/mL and 80 μg/mL, the GSH levels in MPP^+^-induced SHSY5Y cells were significantly restored (Figure 6A). The MDA content was reduced (*p* < 0.01) (Figure 6B). The results show that CP alleviates the oxidative stress level of SH-SY5Y cells induced by MPP^+^. WB analysis was employed to assess protein expression levels in different groups. Compared to the control group, the levels of Nrf2 and HO-1 were significantly decreased. At the same time, the expression of iNOS was increased dramatically in the model group (Figure 6D). After CP treatment, the levels of Nrf2 and HO-1 were considerably restored, and the expression of iNOS was significantly reduced. These findings suggest that CP reduces apoptosis and oxidative stress in MPP^+^-induced SH-SY5Y cells through the Bcl-2/Bax/Caspase-3 and Nrf2/HO-1 pathways, thereby exerting a neuroprotective role.

## 3. Discussion

TCM encompasses more than botanical remedies. TCM comprises four main categories: botanical, fungal, animal, and mineral medicines [26]. Among these, animal-derived medicines often exhibit potent therapeutic effects with reduced toxicity and side effects [27]. CP is the cast-off skin of Cryptotympana pustulate Fabricius. It is a commonly used Chinese medicine recorded in the “Mingyibielu” [28]. Modern studies have demonstrated CP’s diverse pharmacological effects, but there are few studies on treating PD. *N*-acetyldopamine, abundant in CP, exhibits anti-inflammatory and antioxidant activities [29,30], suggesting that these compounds may be the main active ingredients of CP for its therapeutic effects [31], which deserve further study. Our findings demonstrated that CP significantly reduced SH-SY5Y cell damage, inhibited apoptosis, and enhanced mitochondrial homeostasis in an MPP^+^-induced cell model. Animal experiments further verified CP’s anti-PD efficacy, revealing significant improvements in motor dysfunction and activity levels in MPTP-induced PD mice. IF and WB analyses indicated that CP treatment restored TH neuron levels in the SN, protected dopaminergic neurons from MPTP-induced damage, and reduced α-syn accumulation in the striatum.

Metabolomics enables precise exploration of metabolic mechanisms, offering critical insights for this study [32,33]. Using non-targeted serum metabolomics, we identified ten endogenous biomarkers significantly modulated by CP treatment in PD mice, including tyrosine, DA, lysine, L-carnitine, 2-hydroxyoctanoic acid (2-HA), etc. CP improved the amino acid metabolism, as evidenced by increased serum levels of tyrosine, lysine, serine, and isoleucine. Notably, CP enhanced DA levels, protecting dopaminergic neurons and mitigating metabolic disturbances [34]. Overall, our metabolomic analysis supports the effective ameliorative effect of CP on MPTP-induced PD. The MetaboAnalyst analysis revealed that these metabolites are primarily involved in the amino acid metabolism and fatty acid β-oxidation, both closely linked to oxidative stress [35,36]. This conclusion has strong support and reference values for subsequent pathway research. Consistently, our findings demonstrate that CP effectively mitigates oxidative stress and apoptosis in MPP^+^-induced SH-SY5Y cells by modulating the Bcl-2/Bax/Caspase-3 and Nrf2/HO-1 pathways, thereby exerting a neuroprotective role.

This study also has some limitations. Firstly, in the future, we will conduct a more comprehensive evaluation of the efficacy of CP, including long-term safety evaluations or toxicity studies, and compare the efficacy with existing PD therapeutics such as dopamine agonists. Secondly, there are many limitations in translating the effects of CP in the MPTP-induced PD model to human patients, which should be further considered in subsequent clinical trials. In addition, CP focuses on oxidative stress and apoptosis mechanisms in treating PD. Given this, CP has the potential to be applied to other neurodegenerative diseases related to oxidative stress and apoptosis, such as AD and Huntington’s disease. Finally, in the future, we will analyze the chemical composition of CP to find effective compounds that play an anti-PD role, deeply explore the mechanisms of action of CP’s effective ingredients in the treatment of PD, and provide a theoretical basis for the modernization of TCM.

## 4. Materials and Methods

### 4.1. Cell Experiments

#### 4.1.1. Preparation of Ethanol Crude Extract of CP

CP was purchased from Bozhou Shanshengtang Technology Co., Ltd., Bozhou, China. The CP was washed, crushed, and refluxed with 70% ethanol two times: the first time with 8 times the amount, the extraction time was 2h; the second time with 6 times the amount, the extraction time was 1.5 h. The two medicinal solutions were combined and evaporated in a water bath, and the extract was obtained [16], which was freeze-dried (the yield was 8.60%). The powder was stored at 4 °C for later use.

#### 4.1.2. Cell Culture and Treatment

The SH-SY5Y neuroblastoma cell line was obtained from the Boster Biological Technology Co., Wuhan, China. The cells were cultured in DMEM containing 10% fetal bovine serum and 1% penicillin–streptomycin antibiotics (41401ES76, Yeasen Bio, Shanghai, China) at 37 °C, 5% CO_2_, and 95% saturated humidity. The SH-SY5Y cell density was adjusted to 1 × 10^3^ cells/mL and inoculated in 96-well plates at 100 μL/well. The cells were treated with the control group, model group, CP low-dose group (20 μg/mL), CP medium-dose group (40 μg/mL), and CP high-dose group (60 μg/mL). The cells were treated with 0.8 mM MPP^+^ iodide (DMV758, Hangzi Bio, Guangzhou, China) diluted in dimethyl sulfoxide (DMSO) to induce a PD-like phenotype for 24 h.

#### 4.1.3. Measurement of Cell Viability

The cells were treated with different groups of drugs 24 h after MPP^+^ treatment, and a 3-(4,5-dimethylthiazol-2-yl)-2,5-diphenyl-2H-tetrazolium bromide (MTT) assay (A600799, Sangon Biotech, Shanghai, China) was used to evaluate cell viability and after adding 20 μL of 5 mg/mL MTT to each well and cultured for 4 h at 37 °C. Then, 100 μL of DMSO was added to each well and shaken for 10 min to dissolve the crystals fully. The absorbance (OD) value of each well at a wavelength of 570 nm was measured on an ELISA reader (EPOCH2NS-SN, BioTek, Winooski, VT, USA) and the OD value was used as a parameter reflecting the activity of SH-SY5Y cells. Cell survival rate = (OD value of the experimental group − background OD value)/(corresponding OD value of the control group − background OD value) × 100%. Values and standard deviations are from 3 independent experiments.

#### 4.1.4. MitoTracker Assay

The cells were inoculated in six-well plates and treated with different methods for 24 h before the culture medium was discarded and washed with PBS three times. The cells were treated with 0.1 μg/mL MitoTracker™ Red CMXRos (Beyotime, Shanghai, China) and incubated in the dark for 15 min. After washing with PBS, the films were observed under a fluorescence microscope (OLYMPUS IX73, Tokyo, Japan).

#### 4.1.5. Hoechst 33342 Assay

The cells were inoculated in six-well plates and treated with different methods for 24 h. They were washed with PBS three times, and freshly prepared 95% paraformaldehyde was added to fix them for 10 min. After washing with PBS, the cells were treated with 10 μg/mL Hoechst 33342 (Yeasen Bio, China) and incubated in the dark for 15 min. After carefully washing with PBS, the cells were observed under a fluorescence microscope (OLYMPUS IX73, Japan).

### 4.2. Animal Experiments

#### 4.2.1. Animals and Drug Administration

All experiments and operations complied with the relevant management regulations for experimental animals and were approved by the Ethics Committee. All male C57BL/6 SPF mice (34 ± 5 g, provided by the Shanghai Institute of Materia Medica, Chinese Academy of Sciences) were adapted at 22 ± 2 °C, humidified 50 ± 5%, and the 12 h light–dark cycle of the environment for 7 days. All mice were pre-trained for 3 days and then randomly divided into four groups (*n* = 10): the control group, the MPTP group, MPTP+CP low-dose group (10 mg/kg), and MPTP+CP high-dose group (25 mg/kg). After continuous administration for 5 days, the control and MPTP groups were given saline. Starting from the 8th day, 1 h after gavage, all experimental mice except the control group were subcutaneously injected with MPTP (S0620A, Meilunbio, Dalian, China) 30 mg/kg/d, and the control group was given the same volume of saline for 4 days. Behavioral testing began on the 12th day, and the mice were killed after all tests. The experimental process is shown in Figure 2A.

#### 4.2.2. Pole Test

A pole test was used to evaluate the motor coordination and balancing ability of mice. A straight wooden pole with a diameter of 2.5 cm and a length of 100 cm was made and placed vertically on the ground. The mouse was placed head down on the top of the pole, and the time it took to climb from the top to the bottom of the pole was recorded. The mice were pre-trained for 3 days before the experiment. The differences in motor coordination and balance ability of each group of mice were analyzed based on the time each group took to reach the bottom of the rod.

#### 4.2.3. Rotarod Test

The rotarod test was performed on the mice by using the Rotamex-5 RotaRod (Columbus, America, Shanghai Dashen Industrial Co., Ltd., Shanghai, China) to evaluate their ability to move and balance. The mice were pre-trained for 3 days before the experiment. The time it took for the mouse to fall off the rotating rod was recorded, with a maximum of 120 s. The experiment was conducted three times, with an interval of 15 min each time, and the average of the three experiments was taken.

#### 4.2.4. Open Field Test

The experiment was carried out using a rat spontaneous activity box. The mouse was placed in the center of a 40 cm × 40 cm × 35 cm open field box (20185724, Noldus, Beijing, China), and its free movement was observed. The total distance traveled in the observation area was counted using Noldus’ EthovisionXT 15.0 software to observe whether there were differences in exercise capacity between the groups. Before the next experiment, the device was cleaned with a 75% alcohol solution to eliminate the smell of the previous mouse.

#### 4.2.5. Preparation of Tissue

After all behavioral experiments were completed, mice were anesthetized, perfused transcardially with pre-chilled PBS, and then fixed with pre-chilled 4% PFA in 0.1 M phosphate buffer. All whole brain tissues were immediately removed and placed in a fixative containing 4% PFA at 4 °C overnight. The brain tissues were then embedded in paraffin. Then, 50 μm thick coronal sections were cut continuously on a microtome (RM2235, Leica, Wetzlar, Germany) for immunofluorescence studies. In addition, mouse midbrain and STR tissues were obtained, quenched with liquid nitrogen, and stored at −80 °C for subsequent WB studies.

#### 4.2.6. Tyrosine Hydroxylase (TH) Immunofluorescence Staining

Mouse brain sections in Substantia Nigra (SN) were washed three times with PBS and blocked with 5% normal goat serum for 1 h at room temperature. The sections were then incubated overnight with the primary antibody (1:1000 TH) solution at 4 °C. After primary antibody incubation, the sections were washed three times with PBS and then incubated with the corresponding fluorescently labeled secondary antibody for 1 h at room temperature. After secondary antibody incubation, the sections were washed thrice with PBS, stained with DAPI (19E29C77, Boster, Biological Technology Co., Wuhan, China) for 10 min, and observed with a confocal laser scanning microscope (PANNORAMIC MIDI II (3D Histech, Budapest, Hungary)).

#### 4.2.7. Immunoblotting Assay

Protein lysis buffer was added to the animal’s midbrain and striatum tissues, the brain tissue was homogenized, the supernatant was taken, and the protein was quantified using a BCA kit (P0010, Beyotime, China). After the protein was separated by 10% sodium dodecyl sulfate-polyacrylamide gel electrophoresis (SDS-PAGE), it was transferred to a polyvinylidene fluoride (PVDF) membrane (Immobilon-PSQ, K4SA1716B, Darmstadt, Germany). After the membrane was transferred, it was blocked with 5% skim milk powder at room temperature for 1 h, and the primary antibody (1:1000 α-syn [S-441-86], 1:1000 TH [ET1611-12]) was added and incubated overnight at 4 °C. The next day, the secondary antibody of the appropriate host was added and incubated at room temperature for 1–2 h. After the incubation, the membrane was washed, the ECL substrate (Yeasen, Shanghai, China) was added, and it was placed in a chemiluminescence imager for development. Finally, ImageJ software 1.53m (NIH, Bethesda, MD, USA) was used to analyze the protein band intensity.

### 4.3. UPLC-MS Metabolomics Analysis

#### 4.3.1. Sample Preparation

After the last administration, the mouse blood was collected in a centrifuge tube, left to coagulate for 30 min, centrifuged at 8000 rpm for 5 min, and the supernatant was collected and frozen at −80 °C. Pipette 50 μL of sample into an Eppendorf tube, add 200 μL of extract; vortex mix for 30 s, ultrasonicate for 10 min; let stand at −40 °C for 1 h; centrifuge the sample at 4 °C, 12,000 rpm for 15 min; take the supernatant into the injection bottle for detection; and take an equal amount of supernatant from all samples and mix them into control group (QC) samples for detection.

#### 4.3.2. UPLC-Orbitrap/MS Analysis

LC-MS/MS analyses were performed using the UHPLC system (Vanquish, Thermo Fisher Scientific, Waltham, MA, USA) with a Waters ACQUITY UPLC BEH Amide (2.1 mm × 50 mm, 1.7 μm) coupled to an Orbitrap Exploris 120 mass spectrometer (Orbitrap MS, Thermo Fisher Scientific, Waltham, MA, USA). The mobile phase consisted of 25 mmol/L ammonium acetate and 25 mmol/L ammonia hydroxide in water (A) and acetonitrile (B). The auto-sampler temperature was 4 °C, and the injection volume was 2 μL. The acquisition software continuously evaluates the full-scan MS spectrum in this mode. The ESI source conditions were set as follows: sheath gas flow rate as 50 Arb, Aux gas flow rate as 15 Arb, capillary temperature 320 °C, full MS resolution as 60,000, MS/MS resolution as 15,000, collision energy: SNCE 20/30/40, spray voltage as 3.8 kV (positive) or −3.4 kV (negative), respectively.

#### 4.3.3. Data Analysis

A principal component analysis (PCA) and orthogonal partial least squares-discriminant analysis (OPLS-DA) were used for data analysis, and the permutation test was used to test the model. Endogenous differential metabolites were screened according to the projection importance VIP > 1 and *p* < 0.05 and were identified according to the HMDB (http://www.hmdb.ca, accessed on 7 August 2024) and KEGG (http://www.kegg.jp, accessed on 9 August 2024) online databases. MetaboAnalyst 6.0 (https://www.metaboanalyst.ca, accessed on 14 September 2024) was used to perform metabolic pathway enrichment analysis on metabolites.

### 4.4. Mechanism Analysis

#### 4.4.1. Determination of Glutathione (GSH) and Malondialdehyde (MDA) Levels in Cells

Reduced GSH contents in the cells were measured using a chemical assay kit (S0053, Beyotime, China) following the manufacturer’s instructions. The level of MDA was measured using the xanthine oxidase assay and thiobarbituric acid assay in SH-SY5Y cells, respectively. A BCA kit (P0010, Beyotime, China) detected the protein content according to the manufacturer’s instructions.

#### 4.4.2. Pathway Verification

The cell tissues were lysed and proteins were extracted. According to the instructions, protein quantification was performed using a BCA kit (P0010, Beyotime, China). Proteins were separated by 10% sodium dodecyl sulfate–polyacrylamide gel electrophoresis (SDS-PAGE) and transferred to polyvinylidene fluoride (PVDF) membranes. After the transfer, the membranes were blocked with 5% skim milk powder at room temperature for 1 h and then incubated with primary antibodies (1:2000 Bax[ET103-34], 1:2000 Bcl-2[ET1702-53], 1:1000 cl-caspase 3[HA2267], 1:1000 Nrf2[HA721432], 1:1000 HO-1[HA721854], and 1:1000 iNOS[JE59-61]) at 4 °C overnight. The next day, the membranes were incubated with secondary antibodies of appropriate hosts at room temperature for 1–2 h. After the incubation, the membranes were washed. ECL substrate (Yeasen, Shanghai, China) was added, and the membranes were placed in a chemiluminescence imager (Bio-red Gel doc XR+, Bio-Rad Laboratories, Hercules, CA, USA) for development. Finally, ImageJ software was used to analyze and measure the protein band intensity.

### 4.5. Statistical Analysis

All statistical analyses were performed using GraphPad Prism 9 (San Diego, CA, USA). Student *t*-test and one-way ANOVA were used to compare the groups. All data are presented as mean ± standard deviation (S). *p* values < 0.05 were considered statistically significant, with * indicating *p* < 0.05, ** indicating *p* < 0.01, *** indicating *p* < 0.001, and **** indicating *p* < 0.0001.

## 5. Conclusions

This study reveals the pharmacodynamic mechanisms of CP in treating PD by comprehensive in vivo, in vitro, and metabolomics analyses. The results show that CP significantly reduced SH-SY5Y cell damage, inhibited apoptosis, and enhanced mitochondrial homeostasis in an MPP^+^-induced cell model. In vivo experiments, CP significantly improved movement disorders in MPTP-induced PD in mice, protected dopaminergic neurons from damage, and reduced the aggregation of α-syn in the striatum. The metabolomics results show that CP significantly regulated the metabolic disorders of ten biomarkers, mainly involving the amino acid metabolism, which is closely related to the oxidative stress pathway. Further pathway validation confirmed that CP may exert neuroprotective effects through the dual signaling pathways of Bcl-2/Bax/Caspase-3 and Nrf2/HO-1. These findings emphasize the potential of CP as a promising candidate for treating PD, highlight its neuroprotective effects, and strengthen its value in drug repurposing strategies.

## Figures and Tables

**Table 1:** The differential metabolite associated with PD in serum detected using UPLC-MS.

Metabolite	Mode	Retention Time(s)	*m/z*	Formula	Trend
2-HA	NEG	41.8	159.1025	C_8_H_16_O_3_	down
3-HPA	NEG	62.7	165.0555	C_9_H_10_O_3_	down
Isoleucine	NEG	161.8	130.0872	C_6_H_13_NO_2_	up
Betaine	POS	165.3	118.0857	C_5_H_11_NO_2_	up
Methionine	NEG	172.5	148.0436	C_5_H_11_NO_2_S	up
Taurine	NEG	177.2	124.0072	C_2_H_7_NO_3_S	up
S1P(d18:1)	POS	177.7	380.2549	C_18_H_38_NO_5_P	up
Valine	NEG	180.3	116.0716	C_5_H_11_NO_2_	up
Tyrosine	POS	182.2	182.0805	C_9_H_11_NO_3_	up
Dopamine	POS	182.2	136.0752	C_8_H_11_NO_2_	up
Proline	NEG	185.4	114.0560	C_5_H_9_NO_2_	up
Threonine	POS	207.2	120.0651	C_4_H_9_NO_3_	up
Carnitine	POS	208.7	162.1118	C_7_H_15_NO_3_	up
UTP	POS	218.2	484.9706	C_9_H_15_N_2_O_15_P_3_	up
Serine	NEG	220.4	104.0352	C_3_H_7_NO_3_	up
Sn-Glycerol 3-phosphate	NEG	249.4	171.0063	C_3_H_9_O_6_P	up
Ornithine	POS	291.5	115.0862	C_5_H_12_N_2_O_2_	up
Arginine	POS	297.5	175.1183	C_6_H_14_N_4_O_2_	up
Lysine	POS	298.1	147.1124	C_6_H_14_N_2_O_2_	up

## Data Availability

The original contributions presented in this study are included in the article/Supplementary Materials. Further inquiries can be directed to the corresponding author(s).

## Supplementary Materials

The supporting information can be downloaded at: https://www.mdpi.com/article/10.3390/ijms26020544/s1.

## Abbreviations

CP, Cicadae Periostracum; PD, Parkinson’s disease; IF, Immunofluorescence; WB, Western blotting; α-syn, α-synuclein; STR, striatum; DA, Dopamine; TH, Tyrosine hydroxylase; SN, Substantia Nigra; PCA, Principal component analysis; OPLS-DA, orthogonal partial least squares–discriminant analysis; GSH, glutathione; MDA, malondialdehyde.
