# Supplementary material for: Pharmacodynamic Mechanisms of Cicadae Periostracum in Parkinson’s Disease: A Metabolomics-Based Study
Source: Int J Mol Sci. 2025 Jan 10;26(2):544. doi: 10.3390/ijms26020544 (PMC11764672; doi:10.3390/ijms26020544)
Supplement: Supplementary file 1 [file ijms-26-00544-s001.zip › ijms-3393212-supplementary.pdf]

Supplementary material images

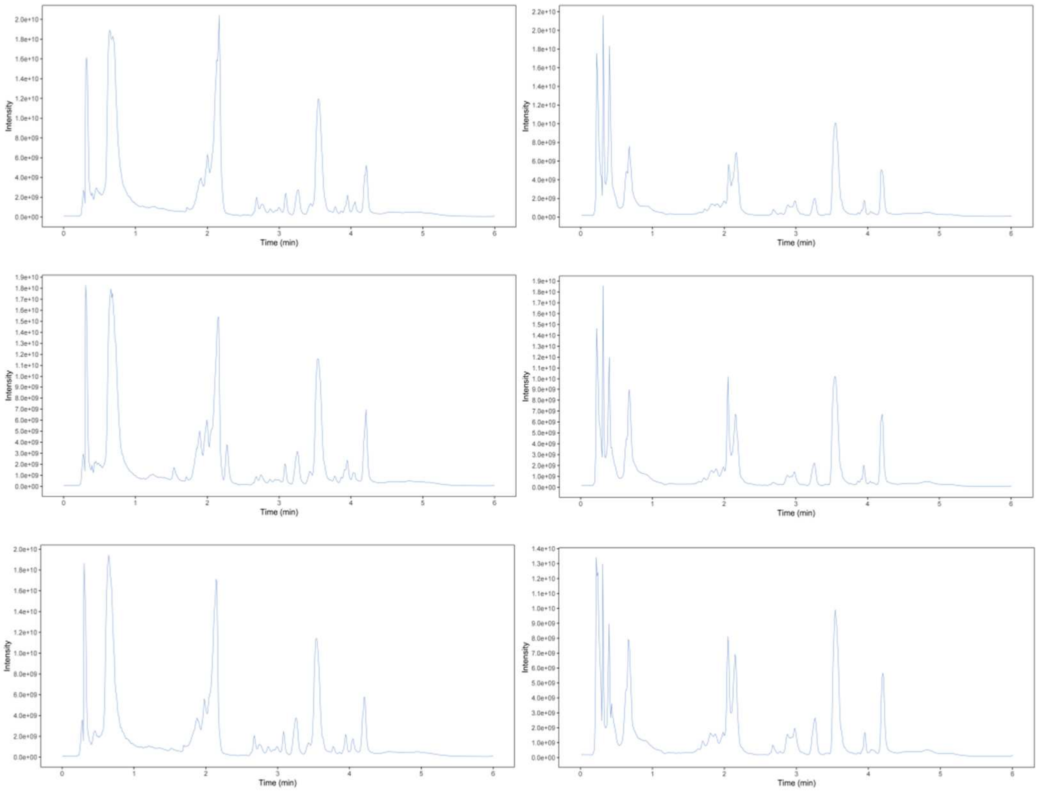

Figure S1. TIC intensity chromatograms of serum samples of control , model and CP groups in the positive (+) and negative (-) ion modes.
